# Early life programming as a target for prevention of child and adolescent mental disorders

**DOI:** 10.1186/1741-7015-12-33

**Published:** 2014-02-24

**Authors:** Andrew James Lewis, Megan Galbally, Tara Gannon, Christos Symeonides

**Affiliations:** 1 School of Psychology, Faculty of Health, Deakin University, Melbourne, Australia; 2 Department of Perinatal Mental Health, Mercy Hospital for Women, Melbourne, Australia; 3 Murdoch Childrens Research Institute, Melbourne, Australia

**Keywords:** Child and adolescent mental health, Developmental origins (DOHaD), Fetal programming, Maternal mental health, Obesity, Prevention, Teterogenic exposures

## Abstract

This paper concerns future policy development and programs of research for the prevention of mental disorders based on research emerging from fetal and early life programming. The current review offers an overview of findings on pregnancy exposures such as maternal mental health, lifestyle factors, and potential teratogenic and neurotoxic exposures on child outcomes. Outcomes of interest are common child and adolescent mental disorders including hyperactive, behavioral and emotional disorders. This literature suggests that the preconception and perinatal periods offer important opportunities for the prevention of deleterious fetal exposures. As such, the perinatal period is a critical period where future mental health prevention efforts should be focused and prevention models developed. Interventions grounded in evidence-based recommendations for the perinatal period could take the form of public health, universal and more targeted interventions. If successful, such interventions are likely to have lifelong effects on (mental) health.

## Introduction

In recent years, a new understanding of the relationship between the early environment and later psychiatric disorder has emerged as the new frontier of psychiatric research. Such research has been largely inspired by the developmental origins of health and disease (DOHaD) model, which proposes a link between fetal development and non-communicable diseases emerging in adulthood such as cardiovascular disease and diabetes [1]. Applying the DOHaD model to research focused on the etiology of mental disorders has yielded some exciting findings. However, translation of these findings to prevent the development of mental disorders has yet to realize the full potential promised by such discoveries. Fundamental to this translational goal is the integration of prevention science, and so this paper aims to provide a review on what may be gained by linking this new understanding of early development to efforts aimed at preventing mental disorders in children and adolescents.

It is well established that the initial onset of high prevalence behavioral and emotional mental disorders frequently commences in childhood or adolescence. Epidemiological studies of population prevalence show rates of child and adolescent mental health disorders to be consistently between 13% and 20% [2-4]. National surveys typically assess common mental disorders in childhood and adolescence across the categories of hyperactive, emotional and behavioral disorders. National prevalence studies in Australia and the US have found that attention deficit hyperactivity disorder (ADHD) is the most prevalent mental health disorder among children and adolescents, followed by emotional and behavioral problems [4,5].

Notably, prevalence rates for mental disorders increase significantly as the child ages and also vary markedly by gender. Gender differences become particularly pronounced in relation to pubertal development. For instance, prior to puberty boys are up to twice as likely as girls to present with hyperactivity and behavioral problems, whereas over the pubertal transition girls become two to three times more likely to present with depressive symptoms than boys [5-8]. The relationship between pubertal development and mental health disorders suggests there may be complex interactions between early developmental platforms established within the first 1,000 days of life and later pubertal development. Gender differences in neurodevelopmental pathways may be programmed early in life via placental and fetal biology but this has attracted relatively little attention as a source of possible gender differences in mental disorders [9].

## Early life programming

The fetal origins of adult disease model was originally proposed by Barker to explain the observed associations between undernutrition of the fetus, low birth weight (defined as birth weight less than 2,500 grams) and an increased risk of cardiovascular disease, diabetes and metabolic syndrome in later life [10]. Low birth weight was initially considered to be the primary indicator of altered fetal development within this model, although other measures of fetal growth later emerged as equally relevant. With the addition of other epidemiological findings such as the role of preconception maternal body composition and under nutrition, as well as the role of processes that do not impact on fetal weight or growth, the model has been expanded to include events beginning prior to conception as well as in early postnatal life. To reflect the developmental aspects of this model, it is now referred to as the DOHaD [11]. The specific programming, in the form of epigenetic programming, cell distribution, and establishment of endocrine systems and metabolic activity, is thought to vary according to the timing, type, dosage and duration of various environmental exposures across early development - now often referred to as the first 1,000 days of life [12].

The DOHaD model draws on a number of evolutionary concepts, the broadest of which is the notion of life history. This idea suggests that the timing of development is a target of genetic adaption such that, across generations, species will adapt to produce the optimal timing and duration for the development of their reproductive life course [13]. The timing and duration of life history traits such as birth, puberty, first reproduction, gestation and onset of senescence, as well as rate of fetal growth and number and size of offspring are all subject to selection pressure [14]. However, within the individual’s life course, greater flexibility is required so each member of a species displays some capacity for developmental plasticity, adjusting the course and timing of development to match prevailing environmental conditions [15-17]. In theory, multiple phenotypes could be produced during development from a single genotype and epigenetic mechanisms are thought to underpin such developmental plasticity [18]. For instance, DNA methylation discordance in epigenomic profiles across a number of different tissues has been observed within monozygotic twin pairs, although dizygotic pairs show an even greater amount of discordance [19]. Developmental plasticity suggests that the capacity to respond adaptively to future environmental conditions enhances the chances of survival and reproductive fitness. This suggests that development is a process where an organism is not only responding to current environmental conditions, but it is also using such information to predict future environmental conditions. Earlier periods of development in the fetal and early infancy period provide indications of the most likely future conditions at a time when there is the greatest degree of plasticity in development. So it follows that the fetal period is regarded as a crucial determinate of whether an organism sets its developmental pathway according to expectations of adversity, stress and high challenge, or develops with expectations of a more benign environment [11].

Fetal programming, therefore, refers to the way in which environmental events alter the course of fetal development, resulting in enduring modifications in the structure and function of biological systems. Programming refers to the influence of a specific environmental factor at a specific point in development, which creates an enduring effect that may result in a bias towards a certain response to subsequent environmental inputs at a later point in development. The work of Meaney and colleagues has been informed by the analogous concept of hormonal imprinting, and they have mapped the role of hormonal signals operating in pregnancy or early postnatal interactions that are able to alter the sensitivity of certain target tissues, often via altered expression of hormone receptors, to these same hormones in later development [20]. It should be noted, however, that the life course of each species will be shaped to emphasize some periods as more or less critical than others. The human life course, for example, has been shaped by a trade-off between bipedalism and gestational length such that the neonatal and early infancy period is one of high vulnerability and plasticity. Furthermore, human puberty as the immediate precursor to reproductive maturation is another period of major biological programming.

These concepts developed within the DOHaD framework can also be used to inform a novel model of vulnerability to mental disorder. Disease and disorder is conceptualized in the DOHaD model in terms of a mismatch between early programming and later environmental conditions. Depending on later environmental inputs, what may have been ‘adaptive’ in the intrauterine environment may prove to be the basis of disease in a future postnatal environment. Equally, the early programming of behavior may prove to be a poor fit to later psychosocial norms, educational expectations or interpersonal demands. In the context of such a model, the psychiatric concept of disorder should be considered not simply as a pathological deviation from normality but as a mismatch across development between early programming and later attempts to adapt to prevailing environmental conditions [10]. Schlotz *et al.* applied the mismatch concept to ADHD, for example. They noted that, in an ancestral environment, early developmental indicators that the environment was going to be harsh or rapidly changing would encourage development of a vigilant individual who is ready to respond quickly to new stimuli and so be better adapted to an unpredictable environment [21]. In a modern context, when an individual developing along this trajectory is placed in a modern educational environment that demands long periods of sustained attention and high levels of concentration, the ‘response ready’ phenotype would be maladaptive.

The DOHaD model is increasingly informed by the emerging understanding of the epigenetic processes that program fetal development. Epigenetic processes do not alter the nucleotide sequence but are responsive to cues from both genes and the environment. Epigenetic programming of fetal and infant development is extremely complex but it does appear that certain exposures can alter epigenetic programming. The most frequently investigated epigenetic processes to date are DNA methylation and histone modification, which play a fundamental role in differentiation of cell structure and function during embryogenesis [22]. Emerging evidence suggests that epigenetic programming continues with significant dynamism across the early postnatal period, with one recent longitudinal study using a genome-wide study of DNA suggesting that one-third of methylation sites show dynamic methylation from birth to 18 months [23] This has led to considerable interest in research examining how altered epigenetic profiles might mediate links between specific intrauterine and early postnatal exposures and future mental health outcomes [22].

## The DOHaD model and child mental health outcomes

An early application of the DOHaD model to mental health outcomes was Barker *et al.*’s use of the Hertfordshire sample to examine rates of adult suicide as a function of birth weight and growth in the first year. They found that birth weight was not itself predictive but that the average weight of 12-month-old infants was over 400 grams lower in cases of suicide [24]. A significant body of research applying the DOHaD model has now examined both birth weight and more specific environmental exposures as predictors of child and adolescent mental disorders [21,25,26].

Since dysregulation of the stress response is a common feature of both emotional and behavioral disorders of childhood and adolescence, mental health researchers have focused much of their attention on factors within fetal development that might impact on the postnatal function of the stress response system. Links between vulnerability to mental disorder and fetal programming of metabolic functioning and immune response have also been investigated but to a lesser extent. Specifically, there has been considerable focus on the early development of the hypothalamic-pituitary-adrenal (HPA) system, which is also linked to maturation of other systems responsible for the regulation of circadian rhythms, physical growth and the integration of limbic-cortical processes. As such, the HPA system plays a critical part not only in stress regulation but also in sleep, feeding, emotions and emotion-regulation [27]. Animal models exposing pregnant mothers to various types of stress or adversity show a clear impact on the development of the offspring HPA system, that manifests in emotional and behavioral disturbances such as fearfulness, impulsivity and substance use. However, it should be noted that the species-specific ontology of the HPA system is important to consider when applying such findings to humans, since exposures at different points in fetal development may influence various systems involved in neuroendocrine and autonomic responses to stressors and the specific timing and degree of fetal HPA development varies considerably across mammalian species.

The development of the ΗΡΑ axis in the human fetus is a complex process involving maturation of fetal organs as well as interaction with placental and maternal endocrine systems [28]. In late pregnancy, a rise in fetal cortisol levels is necessary to stimulate the development of organ systems such as the lungs. However, it seems clear that an excess of fetal glucocorticoids may result in growth restriction of the fetus as well as influencing the postnatal adaptation and activity of the pancreas, pituitary-adrenal axis and cardiovascular activity [29]. Postnatally, an adaptive stress response occurs via perceptual cues relating to threat, disruption of expectancies, physical pain, infection or metabolic crisis. Such cues are communicated to the hypothalamus via specific pathways. These signals are integrated in the hypothalamic paraventricular nucleus, where neurons expressing corticotropin-releasing hormone, in collaboration with other peptides such as vasopressin, stimulate the release of adrenocorticotropic hormone (ACTH) from the anterior pituitary gland [30]. When released into circulation, ACTH stimulates the adrenal cortices to synthesize and release cortisol. The link between pituitary ACTH and adrenal cortisol appears to be established some time after week 20 of gestation [28].

In early gestation, the fetal adrenal cortex produces small amounts of cortisol that gradually increase during the third trimester [28]. Across the second trimester, placental ACTH, in combination with other placental hormones, regulates fetal production of adrenal steroids. By the third trimester, the fetal pituitary gland seems to become integrated with the fetal adrenal cortex [28]. By late gestation, the human fetal HPA axis is well developed and functions as a stress-response system in response to stressors such as hypoxia or nutrient restriction. Therefore, external factors that reduce uterine vascular flow may initiate a fetal stress response similar to that experienced postnatally [31]. Over the third trimester, HPA activation begins to function according to its well-known negative feedback mechanism, whereby mineralocorticoid and glucocorticoid receptors that are expressed extensively across the hypothalamus and hippocampus operate to inhibit the stress response [30]. However, these two receptors play different roles in modulating both the stress response and the circadian rhythm. (Detailed reviews of the HPA system and its fetal development are available in De Kloet *et al.*[30]).

The fetal stress response is rapidly transformed postnatally into a circadian rhythm with a peak around the time of waking and a trough during the day that starts to operate within a few weeks of birth for a term baby [32]. The normal circadian rhythm can facilitate termination of the HPA stress response; conversely, disturbances in the daily rhythm may contribute to HPA stress dysregulation [33]. This postnatal interaction between circadian rhythm, stress response and sleep patterns illustrates how a structure-function relationship established in fetal development can also come to function as the platform for more complex developmental systems. Although these have not been so clearly articulated, similar patterns may exist for the development of interpersonal, emotional and behavioral responses across childhood and adolescence [18].

It follows that placental biology has also been closely linked to the fetal programming of stress response. The placenta functions as a temporary endocrine structure that not only regulates the transfer of nutrients to the fetus but also protects it from the growth-inhibiting effects of maternal glucocorticoids [34]. The placenta serves as a critical interface between maternal and fetal physiology, allowing alterations in maternal endocrine, metabolic and immune systems across pregnancy to interact with fetal development. There is an increasing research focus on the role of the placenta as the link between maternal prenatal distress and infant outcomes. Much of this research has focused on an enzyme (*11β-HSD2*) that specifically inactivates glucocorticoids, is highly expressed within the placenta, and has been suggested to play a role in the ontogeny of the fetal HPA-axis [34,35]. Placental *11β-HSD2* represents a key biomarker of the transmission of maternal stress in pregnancy to the fetus. The placenta may be a central target of maternal pregnancy stress effects and a key mechanistic link between maternal functioning and child mental health outcomes [36].

A number of recent studies are pointing to prenatal maternal stress and depression being key exposures associated with altered epigenetic patterns in both placental tissue and cord blood. These studies provide evidence of alterations in epigenetic programming within specific genes associated with fetal HPA development. Previous studies have used genome-wide DNA methylation scans to examine exposure to psychotropic medications and psychiatric illness in both placenta and cord blood [37,38] and found numerous sites of differential methylation. Alteration in DNA methylation associated with intrauterine exposure to depression has been identified in a number of genes explicitly involved in stress-response systems. For example, Oberlander *et al.* found elevated methylation of the glucocorticoid receptor gene *NR3C1* in cord blood samples from infants born to mothers with depression during the third trimester of pregnancy [39]. In their study, infant HPA reactivity was assessed at three months of age using a measure of information processing designed to induce a mild degree of attention reactivity and, therefore, cognitive stress. Levels of *NR3C1* DNA methylation in fetal cord blood predicted the infant’s cortisol response to this mildly stressful task.

## Major categories of fetal exposure

The quality of the fetal environment can be compromised in several ways. The first is an indirect pathway where physiological reactions to stress such as endocrine, metabolic or immune responses or toxins like nicotine or alcohol produce vascular restrictions, thereby impeding oxygen and nutrition supply to the fetus. The second pathway is a direct transfer of maternal glucocorticoids or other agents across the placenta. In the late 1990s, Nathanielsz summarized three main classes of prenatal exposure that have been investigated for a range of general health outcomes: *lifestyle factors,* such as exercise and nutrition; *maternal mental health,* covering issues of antenatal stress, anxiety and depression; and *teratogenic and neurotoxic exposures* to specific toxins, such as substance abuse, environmental toxins and prescribed medications [40]. Each class of exposure has also been investigated specifically in terms of child and adolescent mental health outcomes and will be used as a guide for the review of such exposures.

### Lifestyle factors

Lifestyle factors such as exercise and nutrition exert a clear influence on maternal and fetal health across pregnancy and have been central to the investigation of fetal programming - mostly looking at cardiovascular and metabolic outcomes. Barker *et al.* noted effects on fetal development of maternal diet based on relatively extreme circumstances such as famine [41]. Maternal diet is critical to offspring growth rates and also has a programming effect on metabolic pathways. These mechanisms are thought to impose lifelong risks for the development of both diabetes and obesity [42].

However, recent work suggests that maternal diet may also exert an influence on the biological systems that underpin future vulnerability to mental disorders [43,44]. Epidemiological evidence suggests that maternal and infant diet influences the risk for both emotional and behavioral disorders in childhood [45]. Jacka *et al.*, for example, reported data from the Mother and Baby study of Norwegian mothers, showing that a higher intake of unhealthy foods during pregnancy predicted behavior problems among children after controlling for a range of confounders. Both maternal nutrition during pregnancy and lactation could be an influence [46].

There is also an emerging body of evidence that maternal obesity during pregnancy is associated with the offspring’s subsequent mental health outcomes. Rodrigues *et al.* found that maternal pre-pregnancy obesity was associated with child inattention symptoms and emotional difficulties [47]. Van Lieshout *et al.* conducted a systematic review of studies on maternal obesity up until 2011 and found that 8 out of 12 studies showed associations between maternal pregnancy obesity and offspring cognitive problems, attention deficit hyperactivity symptoms, eating disorders in adolescence and psychotic disorders in adulthood [48]. Rodrigues suggested that while maternal adiposity at the time of conception may have a programming effect for child mental health, the possible mechanisms remain obscure [47].

Mechanisms for the impact of lifestyle factors can be considered in terms of maternal mechanisms; placental mechanisms, where vascular and metabolic pathways converge in terms of placental function and would expect to be reflected in intrauterine growth retardation as a common pathway; and fetal mechanisms, such as epigenetic changes or differential fetal brain development in response to blood-borne factors that cross the placenta. Fetal pathways would also include fetal counter-regulatory responses to exposures, such as altered blood glucose or lipid ratios, and activation of hormonal signaling molecules such as leptin. While elucidation of the mechanism involved requires further consideration, there are plausible biological pathways involved and this emerging research strongly suggests that a range of lifestyle factors operating across pregnancy appear to influence the child’s subsequent mental health. The relative effects of nutrition, physical activity, obesity and other lifestyle factors are complex and may well interact. However, the evidence appears to be growing that maternal pregnancy and pre-pregnancy lifestyle factors do influence fetal development, and as such would become a modifiable target for prevention intervention.

### Maternal depression and stress during pregnancy

It is well established that children are adversely affected across multiple domains when their mothers’ perinatal mental health is untreated or ineffectively treated [49,50]. Several lines of evidence suggest that perinatal exposure to maternal depression is associated with dysregulation of the child’s HPA response to stress, increasing risk for future stress-related disorders. A wide range of negative child outcomes following maternal depression in the postnatal period have been well documented and these include increased waking cortisol levels during adolescence [51], larger amygdala volume and higher cortisol level at 10 years, higher levels of childhood emotional problems [52], and higher rates of childhood and adolescent depressive symptoms [53]. Infants of depressed mothers show more negative affect and lower sensitivity [54,55] and offspring may experience inadequate physical and verbal stimulation [56].

The global definition of perinatal depression includes both prenatal and postnatal maternal depression and therefore does not allow a clear differential of the effects derived from the intrauterine *versus* postnatal effects. Around 50% of women with postnatal depressive symptoms have also experienced depression during their pregnancy [57,58]. It is well established that postnatal depression reduces the sensitivity of the mother when interacting with her child and this results in poorer stress regulation and insecure attachments. A meta-analysis of seven studies found that the infants of depressed mothers also showed significantly reduced likelihood of secure attachment and raised likelihood of avoidant and disorganized attachment [59]. Essentially, the putative mechanism here is the negative effects of *postnatal* maternal caregiving in the context of maternal depression.

However, a fetal programming pathway for the transmission of maternal prenatal depression to offspring outcome is also a likely contributor that has been relatively neglected by developmental researchers. Animal studies have clearly shown that stress experienced by the mother *during* pregnancy is associated with long-term neurobiological and behavioral effects on her offspring [60]. Studies of prenatal maternal distress in humans show adverse child outcomes, which include symptoms of ADHD [61], lowered cognitive performance and delayed language development [62].

Maternal prenatal stress has an impact on her child’s physiological responsiveness to stress. Specifically, recent studies have found that maternal life stressors during pregnancy predict infant cortisol levels and reactive temperament [63-65], and higher resting cortisol throughout the day in adolescence [66]. It would appear that children born to stressed mothers have higher levels of cortisol, which follows from the disruptions to fetal stress biology previously described. Studies of the relationship between prenatal stress and child mental health have been reviewed recently by Glover [67], van den Bergh *et al.*[68] and Räikkönen *et al.*[69], so here we refer to only a selection of larger studies.

Maternal prenatal stress in various forms is associated with a number of mental health disorders, but most previous research has been based on disaster records or retrospective assessment of prenatal stress. Khashan *et al.*[70] used two Danish national registries and found that maternal prenatal exposure to a family bereavement during the first trimester was related to a 67% increased risk of schizophrenia in offspring after adjustment for demographic confounders. Spauwen *et al.*[71] reported a small increase in risk of psychosis in adolescents whose mothers reported high levels of stress during pregnancy. Kinney *et al.* used data from the national weather service and found that the prevalence of autism spectrum disorder increased markedly with the severity of a storm or hurricane if it was experienced during late pregnancy [72]. Watson *et al.*[73] found that maternal prenatal exposure to a severe earthquake in China was associated with an increased risk of depressive symptoms in offspring, and this risk was more than double for male offspring exposed in the second trimester as compared to female offspring.

A number of large cohort studies have examined maternal anxiety and depression in pregnancy to prospectively predict child mental health outcomes. Loomans *et al.*[74] examined prenatal state anxiety and child outcomes at five years of age in a sample of over 3,000 mothers from the Amsterdam Born Children and their Development study. Maternal state anxiety measured at 16 weeks’ gestation was significantly associated with an increased likelihood of inattention or hyperactivity problems for boys (odds ratio = 2.39) but was not significant for girls. Using the Avon Longitudinal Study of Parents and Children, O’Connor *et al.*[65,75] examined over 7,000 mother-child pairs and found that prenatal maternal anxiety measured at 32 weeks was a significant predictor of inattention or hyperactivity symptoms in boys at 48 and 81 months. However, maternal anxiety measured at 18 weeks’ gestation was not a significant predictor of inattention or hyperactivity scores in boys or girls. Drawing on the Mater University of Queensland Study of Pregnancy, Clavarino *et al.*[76] examined a sample of close to 4,000 mother-child pairs and reported that high prenatal maternal anxiety was associated with an increased risk for attention problems at 5 years that remitted by 14 years (odds ratio = 1.45) and with persistent anxiety problems from 5 to 14 years (odds ratio = 3.02). Robinson *et al.*[77] studied a sample of 1,700 drawn from the West Australian Raine Study. Women were asked at 18 and 34 weeks’ gestation if they had experienced major life stressors and then completed the Child Behavior Checklist when their children were two and five years of age. This study found that a higher number of stressful events was associated with a 23% increased likelihood of behavioral problems at age two and five and a 15% increase in the likelihood of emotional problems at age five.

The bulk of the evidence, and current practice in perinatal mental health, is concerned with addressing maternal antenatal depression and anxiety so as to improve the chances of more effective parenting postnatally. However, the findings emerging from fetal programming research suggest that child stress biology is probably being established during the intrauterine period and that preventions should be focused on preconception and pregnancy mental health and stress exposure of mothers.

### Prenatal teratogenicity and neurodevelopmental toxicity

Prenatal teratogenicity originally referred to the risk of alteration in fetal development resulting in structural changes and malformations in offspring from the use of specific agents in the first trimester of pregnancy. This concept has been expanded to refer to a broader range of exposures across pregnancy, and to outcomes beyond malformations that include longer term child developmental and behavioral outcomes [78]. For an agent to be considered a teratogen there must be a specific mechanism by which that agent alters fetal development, and these effects need to occur with particular timing of exposure during pregnancy and show a dose effect in relation to the outcome of interest [79]. Recent work in environmental chemical exposures highlights the need to consider a broader category of neurodevelopmental toxicants. In particular, low-dose exposure to a number of chemicals with endocrine-disrupting properties is related to adverse neurodevelopmental outcomes in a non-dose-dependent manner [80]. This departure from classical pharmacological models is predicted from the interplay between a complex and tightly controlled endogenous biological system and an exogenous chemical with biological effects outside of normal physiological boundaries. The elucidation of biological pathways does however remain a fundamental step in establishing a convincing case for causality in observed statistical associations between exposure and outcome.

Prenatal teratogenic exposures have been widely investigated, and well-documented associations with increased risk of emotional, behavioral and cognitive problems include environmental neurotoxicants like lead; substances of abuse such as alcohol, cigarettes and cocaine; and prescribed medications, such as the antiepileptic drug sodium valproate. The evidence for effects from other psychotropic medications is less well established [21,81-84]. Cigarette smoking has been found in up to 11.8% of pregnant women [85], with 30.3% of women having some alcohol in pregnancy but only 2.7% having alcohol across all trimesters [86]. Illicit substance use is likely to be lower, as is exposure to antiepileptic drugs. However, exposure to antidepressant medications was found in as many as 13.4% of pregnancies in one study from Tennessee in the US [87]. Environmental chemical exposures, by contrast, can be near ubiquitous, underlining the population significance of even subtle neurodevelopmental toxicity. Consider for example that between 1976 and 1980, 77.8% of the US population had blood lead levels that were more than double the current threshold for reporting [88].

There are established associations between exposure to maternal smoking in pregnancy and a range of pregnancy and child health outcomes, from growth restriction and preterm delivery to childhood respiratory illness. Increased incidence of childhood mental illnesses and symptomatology, specifically ADHD and conduct problems, have also been consistently observed [89-93]. Even environmental tobacco exposure (passive smoking) is associated with adverse behavioral outcomes [94-96], although a causal biological pathway has not been established. An association with childhood anxiety and depressive symptoms has been observed [97], but not consistently [93]. A number of recent studies have raised doubts about whether the observed associations between maternal smoking during pregnancy and childhood mental health are fully causal in nature or reflect, in part, shared genetic susceptibility. In the Avon Longitudinal Study, the effects size for paternal smoking was of similar magnitude to that of mothers [98], and in a study of children born following assisted reproduction, the association between maternal smoking in pregnancy and childhood ADHD symptoms was greater in those where the child was genetically related to the mother [99]. Shared inheritance does not, however, appear to account for all of the observed association between ADHD and prenatal tobacco exposure in other cohorts [91,93,100]; a recent study from Taiwan explicitly tested and found evidence for a biological pathway that is dependent upon tobacco-related chemicals. Hsieh *et al.* used genetic studies of children to demonstrate that the association between cord blood cotinine and childhood behavioral difficulties is modified by a genetic polymorphism in the metabolic pathway for smoking-related toxicants [94].

Alcohol has also been associated with a range of teratogenic effects, from fetal alcohol syndrome to a broader fetal alcohol spectrum and later onset developmental and behavioral problems, such as low IQ, specific learning disorders, and internalizing and externalizing symptoms [101,102]. Of the other drugs of abuse, cocaine [103,104], marijuana, benzodiazepines and methamphetamine [105] have each been associated with effects on neurodevelopment and later child mental health outcomes that appear to be independent of social factors [106,107]. These effects are subtler than earlier research in the area, and are not apparent until much later in child development and therefore referred as latent or ‘sleeper’ teratogenic effects [78]. The evidence for opiate exposure is unclear [103]. Epidemiological research in the area is complicated by covariance between substance use and social factors and, in the case of opiates, the small numbers of pregnancies affected and challenges of follow-up in this relatively chaotic social group.

More recently, longitudinal studies have started to focus on psychotropic medications including antidepressants, antipsychotics and mood stabilizers. Studies from North America have shown an increasing rate of antidepressant exposure in pregnancy, ranging from 7.6% to 13.4% in the US and 5% in Canada [87,108,109]. In Australia, the rate has been shown in data from the Longitudinal Study of Australian Children to be around 2.1% [110]. The difficulty in examining these agents for potential teratogenic effects is untangling the potential impact of the often serious maternal mental illnesses these agents are used to treat as well as co-morbid exposures that confound outcomes. For instance, one study examining malformation risk and antidepressant exposure found fetal alcohol syndrome was 10 times more likely in children exposed to antidepressants in pregnancy [111].

The most rigorously studied psychotropic class is the antiepileptic drugs, which, in addition to being used in epilepsy, are used as mood stabilizers for treatment of bipolar disorder. These agents have previously been associated with an increased risk of specific structural teratogenicity, such as an increased risk of neural tube defects. There are now a number of rigorous, prospective, longitudinal studies that have followed children from pregnancy to school age to examine for neurodevelopmental and behavioral teratogenic effects [112]. They have identified specific risks with exposure to specific agents and a dose effect. The antipsychotic medications, both typical and atypical, are not associated with a malformation risk but the literature for longer term effects is far too limited to draw any conclusions about child development outcomes [113,114].

There are now a number of large studies of antidepressant exposure in pregnancy and malformation risk but there is still no consensus as to whether there is a small increased risk of birth defects [115]. Studies of longer term teratogenic risks are more limited and most have a small number of participants and short follow-up [116]. However, while no studies to date have found an effect of exposure on global cognition, there are four studies that have found an increased risk of poorer motor development [117-120]. It is important to balance these findings with a number of studies that have found that untreated depression is associated with poorer development, particularly language development [121]. Given the increasing rate of exposure to this class of psychotropic medication, further studies are required that can robustly quantify the potential risks of exposure to balance against the harms of withholding treatments. Such studies ideally require longer follow-up, robust consideration of maternal depression and other confounding factors, and more robust child development measures in order to reach clear conclusions [87,108,122,123].

It can be concluded from studies on psychotropic medication that, to minimize effects on longer term child development, single agents should be considered when treating maternal mental illness in pregnancy, keeping doses as low as is feasible for effective treatment. There is an urgent need for further studies to delineate risks for specific agents so that more informed choices can be made [124-126]. When considering the use, and hence exposure, to antidepressants in pregnancy, the issues relevant to child development and mental health outcomes are not just those that relate to exposure *per se* but also to the impact of untreated maternal mental illness. There is mortality data in both the UK and Australia that suggest mental illness is a leading indirect cause of maternal deaths [127]. In addition, untreated depression potentially impacts on the capacity of women to self-care in pregnancy, particularly in important areas that are increasingly associated with optimal fetal growth and development, such as nutrition and exercise. Finally, clinicians and patients need to consider the effects that remaining depressed may have on a woman’s capacity to enjoy motherhood, bond with her baby, and provide responsive and sensitive parenting to the child. All of these aspects of parenting also have a significant impact on short- and long-term child outcomes.

Environmental teratogens and neurodevelopmental toxicants differ from the above by the locus of control of the mother with respect to exposure, but are nevertheless an important modifiable risk factor for preventative mental health strategies. Lead exposure is the prototypic environmental neurotoxicant. Historical exposure was through use of lead in water pipes carrying drinking water, as a fuel additive, and in paints and certain toys. Lead persists in the environment and current household exposure is understood to be primarily from historical soil contamination and old paint, although lead does also continue to be used in a restricted form in hobby activities including soldering, pottery, collectables such as toy soldiers, certain artists’ paints, ammunition and fishing sinkers. Large longitudinal studies demonstrate that prenatal [128-130], lifetime [129,131] and current exposure [132-134] are each important [135-137] for neurodevelopmental outcomes. The proposed biological mechanisms have supportive *in vitro* evidence, including inhibition of N-methyl-D-aspartic acid glutamate receptors (the key molecule regulating synaptic long-term potentiation) and interaction with calcium ion signaling, with much wider implications. Although much research has focused upon outcomes of general cognitive ability and/or ADHD, there is well-replicated evidence for an association between lead exposure and adverse outcomes across broad ranging neurodevelopmental outcomes, including both behavioral and emotional symptomatology [138-141]. The effect size is substantial. Froehlich *et al.* estimate that in the US, 25.4% of ADHD in 8- to 15-year-olds is attributable to the low levels of exposure that persist today [142]. Their analysis used data from the US National Health and Nutrition Examination Survey (NHANES) study, a cross-sectional study but whose design facilitates it having the statistical power to look at clinical outcomes rather than symptomatology.

The evidence base for lead exposure and neurodevelopmental outcomes is not matched anywhere else in the environmental chemical literature. Other environmental chemicals with established neurodevelopmental toxicity include methyl mercury and the polychlorinated biphenyls (PCBs). Environmental exposure to each has established associations with general cognitive function, although the relevance to broader mental health is less clear [143]. Interestingly, the longitudinal data that does exist support an association with prenatal but not postnatal exposure, in support of the DOHaD hypothesis [143]. The strongest evidence is for associations with ADHD and executive function deficits [143]. Wider mental health outcomes have not been adequately studied to draw conclusions, although data would exist in the New Bedford cohort [144,145] that we have not been able to find in publication. Biological mechanisms are also unclear, although PCBs are potent endocrine disruptors and modeling supports thyroid function as a putative mechanism [146].

No consistent specific pattern of deficits emerges that distinguishes neurodevelopmental toxicity due to lead from that due to mercury or PCBs. Observed outcomes in each span both cognitive and affective domains [143], suggesting common developmental pathways of neurodevelopmental toxicity with relevance to mental health. Research to date does imply that cognitive performance and behavioral problems are more strongly associated with environmental toxicity than emotional problems, although it is not clear if this is due to greater sensitivity to neurodevelopmental toxicity, or greater sensitivity in the population-based methodology and assessment tools used to detect subtle effects at the population level.

Exposure to lead and PCBs are on the decline, and methyl mercury exposure appears to be stable [147-150]. Yet there is substantial work still to be done to further reduce exposure to these established neurotoxicants, most notably lead, where the ongoing effects of exposure estimated from the US NHANES data mean that this must remain a key priority in the preventive mental health agenda [142]. At the same time there is a growing body of modern chemicals - not previously evaluated for neurodevelopmental toxicity - that now raise concern as potential neurotoxins in need of further evaluation [143,151]. These include manganese and cadmium - bivalent heavy metal cations like lead and mercury - and many chemicals with endocrine-disrupting actions *in vitro* similar to those of PCBs (for example, bisphenol A, phthalates, organochloride pesticides, organophosphate pesticides, brominated flame retardants and perfluorinated compounds). The robust evaluation of these chemicals is a substantial new opportunity in preventative mental health. However, beyond even this, there is an appreciation that the potential for neurodevelopmental toxicity is unknown for the vast majority of chemicals in ubiquitous modern usage [151], emphasizing that there may be other substantial opportunities for mental health prevention, and that there is a need to prioritize understanding in this area.

Addressing environmental toxins, smoking, alcohol and illicit substance use in pregnancy are important to reduce the implications for child development and mental health outcomes. The latter three also have implications for pregnancy self-care and nutrition. However, there is evidence that both smoking [152] and alcohol use [111] in pregnancy are associated with depression in pregnancy. Therefore, interventions that address a broader approach to a healthy pregnancy may be warranted.

## Positive effects of intrauterine exposures

Whereas most studies focus on negative child developmental outcomes following maternal stress or depression over pregnancy, a number of studies present some positive effects of either stress exposure or prescription of antidepressants. These findings suggest that exposures during pregnancy are complex and need to be carefully considered in terms of the type of exposure, its timing, and potential genetic moderators on child development outcomes where some children may manifest poor outcomes while others may in fact benefit from similar levels of exposure.

For instance, DiPietro *et al.* reported that increased levels of particularly anxiety and stress - but not depressive symptoms - in pregnancy were associated with higher levels of motor development and mental development in children at two years after adjustments for postnatal confounds [153]. Notably the sample consisted of only healthy women with low-risk pregnancies whose levels of anxiety and depression were not in the clinical range and stressors were minimal. Genetic factors also add to the complexity, with some polymorphisms such as those within *SLC6A4* conferring sensitivity to advanced development within positive environments [154]. There is also some evidence that the time of stress exposure within pregnancy may be an important factor in the outcome. By sampling repeatedly across pregnancy, Davis and Sandman showed that exposure to higher levels of cortisol in early pregnancy predicted poorer child development outcomes, whereas higher levels of maternal cortisol in late pregnancy predicted accelerated cognitive development [155]. Such findings suggest that effects of mild stress exposure, possibly towards the later period of gestation, may well confer developmental advantage.

There are now also limited animal and human studies that have shown a beneficial effect on offspring exposed to selective serotonin reuptake inhibitors during early development. One study of rats showed fluoxetine exposure protected rat offspring from the effects of pregnancy stress on adolescent outcomes for both depressive symptoms, as measured by the Forced Swim Test, and increased hippocampal neurogenesis [156]. A second study showed that fluoxetine exposure in rat pups separated from their mothers protected against cell apoptosis of the dentate gyrus of the hippocampus [157]. A study of human neonates showed early speech perception was more advanced in those exposed to selective serotonin reuptake inhibitors than those exposed to controls [158]. Other studies examining global cognitive development in relation to antidepressant exposure and maternal depression have also found a significant effect of maternal depression but not antidepressant exposure on outcomes [159,160]. Given the limited number of studies, these findings must be interpreted with caution but do build a more complex picture of the potential risks and potentially protective aspects of exposure, which also requires consideration of the context in which the exposure to antidepressants occurs.

Findings of positive effects of certain exposure may at first appear confusing or suggest methodological flaws in research. However, some degree of stress is a normal part of life, and increasing levels of glucocorticoids across pregnancy are a normal part of development, serving important maturational functions for the fetus. Given that clinical levels of stress and psychiatric disorders impact on multiple regulatory functions in pregnant women, we can infer that only relatively mild stressors in late pregnancy would be advantageous but further research should investigate this assumption.

## Implications for mental health prevention

There is growing evidence from human studies showing that early exposures to lifestyle factors and maternal mental health are predictive of child behavioral, emotional and learning outcomes. Such exposures appear to alter the developmental trajectory according to subtle programming effects. These effects may impact, for example, on the development of the endocrine response to stress, which can manifest as psychiatric disorder at later points in development, particularly when a child or adolescent is confronted by new challenges. This literature on mental health and lifestyle factors supplements and in many respects expands upon the teratogenic and neurotoxic models of exposure. These exposures have a deleterious impact because they introduce intrauterine conditions which fall outside of biological norms while factors such as maternal stressors or nutrition probably have their impact through inducing different trajectories for development as the fetus attempts to adapt to variation within the intrauterine environment. Factors such as illicit substance exposure, smoking and environmental toxins also have strong evidence of associations with child mental disorders. All three areas covered in this review constitute important areas for prevention efforts to target.

Such evidence is now sufficiently compelling that it calls upon researchers to translate such findings into interventions designed to prevent mental disorders. The need for prevention is particularly acute given that mental health treatment systems are coping poorly with demand for mental health services and typically service around 20% of the population’s clinical need [161]. Prevention science and practice has a vital but somewhat neglected role to play in transforming health, education and community service systems to enable the developmental causes of major sources of mental disorder to be effectively targeted. Population and public health models are increasingly being considered as a vehicle for the prevention of high prevalence mental health disorders in childhood, with a focus on attention, emotional and behavioral disorders. It is becoming clearer that risk factors for such common disorders show considerable cross-over with risk factors for major non-communicable diseases [162]. Optimizing health cannot be detached from optimizing mental health [163].

Already a number of successful programs have been developed, such as nurse visitation in the perinatal period [164]. The Nurse-Family Partnership program, originally trialed in New York, has now been replicated in several different populations and shows ongoing child development benefits up to nine years of age [165]. This program comprises home visits by nurses for first-time, disadvantaged mothers across pregnancy and early infancy. The program has three aims: first, to improve pregnancy health and outcomes; second, to improve child health and development; and third, to help parents plan future pregnancies, complete their education and find work [166]. The Nurse-Family Partnership program is associated with a wide range of beneficial outcomes, but of interest to this review is the significant effect on child outcomes, including educational improvement, as compared to control children [165].

A recent Cochrane review reported emerging evidence to show that current interventions aiming to prevent postnatal depression in women are of benefit [167]. Notably, few of these interventions also examine outcomes for children. There is considerable scope for the development of effective interventions for pregnant women to address not only depression but equally anxiety disorders and high stress exposures [168,169]. Overall, the quality of evidence for existing prevention programs in the preconception, pregnancy and early infancy period is limited, and often target only one of the many deleterious factors that impact on child development.

The ultimate goal of prevention in a mental health context refers to measurably reducing the population rates of mental health disorders by using strategic efforts to address their known causes. Since it often adopts a population perspective, prevention science tends to remain at some distance from research on the neurobiological systems implicated in early development. However, prevention efforts could effectively target immature, developing neurobiological systems. Early experience research is highly applicable to prevention as it suggests ways that not only adverse postnatal experiences but also preconception and intrauterine factors might constitute targets for prevention efforts.

The current review has identified strong evidence for the deleterious impact of maternal mental health and a range of teratogenic and neurotoxic factors. There is also emerging evidence for lifestyle factors such as diet and obesity. Further robust evaluation of psychological and chemical exposures that cross the placenta or impede placental function via compromised maternal health and mental health will continue to create substantial new opportunities in preventative mental health. The general implication of the DOHaD model is that the prevention of compromised fetal development would potentially have long-term health and mental health benefits for offspring.

We have focused on three major classes of pregnancy exposure - not to suggest that these are exhaustive but to provide a framework to guide consideration of intervention efforts. As Schlotz *et al.* note, an interesting feature of the fetal programming research is that the diverse range of exposures reviewed - such as prenatal smoking, stressful events and depression - show broadly speaking similar patterns of outcomes in terms of child behavioral, emotional and attention deficits [21]. This suggests placental transmission or compromised placental function may comprise a small number of mechanisms that interrupt fetal development and therefore result in a similar set of child outcomes. It also shows the commonality between mental health and other health outcomes. Fetal growth, therefore, could continue to be considered a useful index of fetal adversity but also as an outcome for prenatal and preconception intervention to target, particularly for population-level interventions.

Pregnancy care provides a convenient opportunity for health interventions given the high level of engagement with the health delivery system, and this is a key time point to target public health strategies around psychological and physical preparation for pregnancy, smoking, alcohol, diet, and exposure to known teratogen and neurodevelopmental toxins. However, given that 40% or more of pregnancies are unplanned, and given the importance of the preconception period for many factors we have identified here, a broader approach is needed. Effective strategies would therefore be anticipated to combine targeted interventions within pregnancy care, with broader interventions that will reach all women of child-bearing age, or that are effectively population-wide, following the successful model of folate supplementation for prevention of neural tube defects. This is especially relevant for developing strategies towards reducing environmental exposure to chemicals with neurodevelopmental toxicity.

Interventions that recognize and support the formation of a family unit are important to consider as an alternative to focusing intervention only on maternal care. Partner support has consistently been found to be a major predictor of maternal coping with stress and perinatal depression. Policies and practices to support pregnant women in the general population and also efforts to support women at elevated risk are of particular importance. In perinatal psychiatry, the development of antidepressants that do not cross the placenta and therefore do not impact on fetal development are currently in the development phase and, if effective, t could have a significant impact on reducing antenatal maternal depression and anxiety.

Recommendations arising from the current review are threefold. First, to develop recommendations on pregnancy health based on the current evidence base on exposures relevant to optimizing the mental health of offspring. Such recommendations need to be examined in relation to other preventative measures aimed at improving general pregnancy health so as to capitalize on the commonality of risk factors for both child health and mental health outcomes. Second, health promotion programs targeted to both the public and health professionals to encourage full implementation of such recommendations. Third, targeted intervention to high-risk groups, which might take the form of behavior and education programs for young adult women of child-bearing age in high risk groups concerning pregnancy health and infant development. High risk groups include women who in preconception or during pregnancy experiencing major (mental) health difficulties or other disadvantages, major stressors or other adversities, and mothers of infants who are premature, of multiple birth, or have birth complications, low birth weight or failure to thrive.

## Abbreviations

11β-HSD2: type 2 isoform of 11beta-hydroxysteroid dehydrogenase; ACTH: adrenocorticotrophic hormone; ADHD: attention deficit hyperactivity disorder; DOHaD: Developmental origins of health and disease; HPA: hypothalamic-pituitary-adrenal; NHANES: National Health and Nutrition Examination Survey; PCBs: polychlorinated biphenyls.

## Competing interests

The authors declare that they have no competing interests.

## Authors' contributions

AJL designed the review and drafted the manuscript. MG co-designed the review and conducted a review of psychotropic exposures. TG conduced a review of stress and depression exposures. CS reviewed teratogenic and neurotoxic exposures. All authors developed conclusions and recommendations based on the areas reviewed and helped to draft the manuscript. All authors read and approved the final manuscript.
